# Integration of ultra-widefield optical coherence tomography angiography images and clinical indicators for identifying early-stage diabetic retinopathy in type 2 diabetes

**DOI:** 10.3389/fendo.2026.1941613

**Published:** 2026-09-24

**Authors:** Cong Zhang, Li Wang, Shimeng Huang, Ruoxuan Zheng, Jingjing Li, Xiaotong Sun, Shuang Wu, Tianpeng Zhang, Xinyu Zhang, Qian Xu, Yangwen Chen, Chengye Xu, Hongyu Kuang

**Affiliations:** Department of Endocrinology, The First Affiliated Hospital of Harbin Medical University, Harbin, China

**Keywords:** clinical factors, diabetic retinopathy, multimodal deep learning, type 2 diabetes mellitus, ultra-widefield optical coherence tomography angiography

## Abstract

**Background:**

Diabetic retinopathy (DR) is a common microvascular complication of diabetes and a leading cause of irreversible vision loss among working-age adults, making early detection essential for preventing permanent visual impairment. This study aimed to identify early-stage DR in patients with type 2 diabetes mellitus (T2DM) using a multimodal deep learning (DL) approach integrating ultra-widefield optical coherence tomography angiography (UW-OCTA) images with clinical factors.

**Methods:**

This cross-sectional study enrolled patients with T2DM from an urban tertiary hospital and a community primary care setting between July 2024 and April 2026. Baseline demographic and laboratory data and 26 × 21 mm UW-OCTA images were obtained. Automatically segmented UW-OCTA slabs were compared to identify the most discriminative layer. Clinical variables were selected using between-group comparisons, XGBoost-based Shapley additive explanations (SHAP), and multivariable logistic regression. Selected clinical variables and UW-OCTA image features were integrated into a multimodal model. Performance was evaluated in internal test and external validation sets using receiver operating characteristic analysis. Gradient-weighted class activation mapping (Grad-CAM) was used for visualization.

**Results:**

Overall, 1,676 patients were included: 1,047 and 453 tertiary-care patients formed the training and internal test sets, and 176 community-based patients formed the external validation set. Diabetes duration, glycated hemoglobin, low-density lipoprotein cholesterol and systolic blood pressure were independently associated with early-stage DR. The retinal slab showed the best image-only performance with AUCs of 0.906 (95%CI: 0.870-0.942) and 0.828 (95%CI: 0.725-0.931) in the internal and external sets. The multimodal model achieved AUCs of 0.921 (95%CI: 0.887-0.954) and 0.849 (95%CI: 0.760-0.953), outperforming image-only and clinical-only models. Grad-CAM highlighted macular and peripheral retinal regions.

**Conclusion:**

Integrating UW-OCTA with clinical factors improved early-stage DR identification in T2DM and may support non-invasive risk assessment.

## Introduction

Diabetic retinopathy (DR) is one of the most common microvascular complications of diabetes and represents the leading cause of irreversible blindness among working-age adults (1). The prevalence of DR among individuals with diabetes was 22.27%, corresponding to approximately 103.12 million adults with DR in 2020, with this number projected to increase to 160.50 million by 2045 (2). Therefore, early-stage identification of DR is crucial for reducing the risk of vision loss and alleviating the disease burden.

Early-stage identification of DR remains challenging. In its early stage, DR often progresses silently, and patients with early-stage DR may have no obvious visual symptoms (3). However, in the later stage, it progresses rapidly and is highly likely to cause blindness (4). Early-stage detection and timely intervention can substantially reduce the risk of severe visual impairment (5). Nevertheless, with the growing global burden of DR, particularly in settings with limited medical resources, the shortage of ophthalmology specialists makes timely and standardized retinal assessment for many patients with diabetes (2).

As artificial intelligence (AI) continues to advance in medicine (6, 7), it offers new opportunities for the early detection of diabetic retinopathy (DR) (8). Studies have shown that deep learning (DL) applied to fundus photographs can achieve accuracy comparable to retinal specialists (9, 10). Compared with conventional fundus photography, ultra-widefield optical coherence tomography angiography (UW-OCTA) can cover a broader retinal area and enables non-invasive visualization of the retinal microvasculature (11), facilitating AI-based detection of early-stage microvascular abnormalities. Nevertheless, studies on the diagnosis of early-stage DR using UW-OCTA remain scarce. In addition, early-stage DR is influenced by systemic metabolic factors. Evidence from large clinical studies have shown that diabetes duration, glycemic control, blood pressure, and lipid metabolism abnormalities are important factors associated with DR (12), supporting their incorporation as complementary clinical features in AI-based models. Accordingly, integrating UW-OCTA imaging information with key clinical indicators may improve the comprehensiveness and accuracy of early-stage identification of early-stage DR.

Therefore, this study integrates UW-OCTA imaging and key clinical factors to develop a multimodal model for the identification of early-stage DR which may contribute to reducing vision loss and enhancing patient quality of life.

## Methods

### Study design and participants

This cross-sectional study enrolled patients with T2DM who attended an urban tertiary medical institution (the First Affiliated Hospital of Harbin Medical University) and a community-based primary care institution (Hulan District Community Health Service Center) between July 2024 and April 2026. The same predefined eligibility criteria, DR grading criteria, and image-quality requirements were applied to participants from both institutions.The cohort from the urban tertiary medical institution was used for model development and was divided into training and internal test sets at a ratio of 7:3. The right eye was selected *a priori*, with one eligible examination per participant included and all participant-level imaging and clinical data retained within a single cohort Clinical feature selection and model development were performed exclusively using the training set. The cohort from the community-based primary care institution served as an independent external validation cohort to evaluate the generalizability of the model across populations and healthcare settings. All participants underwent ophthalmic examination and retinal imaging assessment. DR status and severity were independently graded by two ophthalmologists based on fundus photographs according to the Early Treatment Diabetic Retinopathy Study (ETDRS) grading criteria (13). The graders were masked to UW-OCTA findings and model predictions. Disagreements between the two graders were adjudicated by a third ophthalmologist to establish the final reference diagnosis. Intergrader agreement between the two primary graders was assessed using Cohen’s κ (κ = 0.904). Mild non-proliferative DR was defined as early-stage DR and was used as the target condition for identification in the final model analysis in our study.

Participants were eligible if they were aged 18 years or older, had type 2 diabetes diagnosed according to the American Diabetes Association Standards of Care in Diabetes (14), and completed UW-OCTA examination. The exclusion criteria were as follows: other retinal diseases that could affect image interpretation; previous ocular treatment that could alter retinal vascular morphology; severe media opacity; poor UW-OCTA image quality; or missing key clinical variables, such as age, sex, or medical history (**Supplementary Table 1**). The study protocol was approved by the Ethics Committee of the First Affiliated Hospital of Harbin Medical University (approval number: 2025XS162-01) and was conducted in accordance with the Declaration of Helsinki. Written informed consent was obtained from all participants.

### Clinical data collection

Clinical data were collected from structured electronic medical records, standardized clinical assessments, and laboratory databases. The collected variables included demographic characteristics, lifestyle-related factors, diabetes duration, history of hypertension (HBP), body mass index (BMI), routine blood parameters, liver and renal function markers, glycemic indices, and lipid profiles. Laboratory measurements were performed in the clinical laboratories of the respective centers according to standardized procedures.

### OCTA image acquisition and preprocessing

All participants underwent bilateral 26 × 21 mm UW-OCTA imaging using a swept-source OCT/OCTA system (VG200S; SVision Imaging, Henan, China; software version 2.1.016). The system used a swept-source laser with a central wavelength of approximately 1050 nm and a scanning speed of 200,000 A-scans per second. Only right-eye images were included in the model analysis. Layer segmentation was performed using the built-in segmentation software (SVision Imaging, version 1.32.9), and automatically segmented en face OCTA slabs, including the retinal layer (RL; full-thickness retinal OCTA slab), superficial capillary plexus (SCP), intermediate capillary plexus (ICP), and deep capillary plexus (DCP), were exported for layer-specific model development. The RL represented the full-thickness retinal slab extending from the internal limiting membrane (ILM) to Bruch’s membrane (BM). The SCP mainly corresponded to the vascular complex between the ILM and the ganglion cell layer, whereas the ICP and DCP were located at the inner and outer sides of the inner nuclear layer, adjacent to the inner and outer plexiform layers, respectively (15). All OCTA images underwent standardized quality control. Images with a quality score lower than 7/10, obvious motion artifacts, poor centration, severe media opacity, image obstruction, or uncorrectable segmentation errors were excluded. Qualified images were resized to 224 × 224 pixels, intensity-normalized, and converted into a standardized format for DL model input. The same deterministic preprocessing procedure was applied identically to images from the tertiary-care and community cohorts.

### Clinical feature selection and model construction

Clinical feature selection was performed exclusively within the training set. A total of 30 candidate clinical variables, defined and available before model development, were initially evaluated. Participants were classified into the early-stage DR group and the non-DR group according to ophthalmic grading. Clinical variables with significant between-group differences (P < 0.05) were entered into an XGBoost model, and Shapley additive explanations (SHAP) analysis was used to evaluate feature importance. To reduce potential collinearity and variable redundancy, candidate variables were further selected according to clinical relevance and SHAP-based importance ranking. A binary logistic regression model was then used to assess the independent associations between the selected clinical variables and early-stage DR. Variables independently associated with early-stage DR were used to construct the clinical model.

### Development of OCTA Image-Based and Multimodal Deep Learning Models

Qualified UW-OCTA images were used to develop image-based DL models. Single-image models were trained separately for each automatically exported UW-OCTA layer to identify the most informative imaging input. Images were preprocessed as described above, with additional data augmentation applied only to the training set using random rotations of up to ±30°and horizontal flipping. The UW-OCTA image-based model was based on a ResNet-18 architecture initialized with ImageNet pretrained weights, with the final layer replaced by a two-class classifier for distinguishing early-stage DR from non-DR. The models were trained end-to-end using class-weighted cross-entropy loss (NDR: early-stage DR = 1:2) and the AdamW optimizer. For the multimodal model, normalized clinical variables selected from the clinical feature selection procedure were processed through a fully connected clinical branch and mapped to 512-dimensional feature representation, while UW-OCTA images were processed through the ResNet-18 image branch to obtain a 512-dimensional image representation. Specifically, the four clinical variables were first projected from a 4-dimensional input into a 64-dimensional feature space through a fully connected layer, followed by ReLU activation, and were subsequently mapped to a 512-dimensional representation. The extracted clinical and image features were fused at the feature level by element-wise addition and subsequently passed through a classifier comprising a 256-unit fully connected layer with ReLU activation and dropout (0.3), followed by the final two-class output. The class with the higher softmax probability was assigned as the final classification result. All models were implemented in Python 3.10.6 using PyTorch 2.11.0, with CUDA 12.8, and were trained on an NVIDIA GeForce RTX 5050 GPU. Additional implementation and training details for the OCTA image-based and multimodal deep learning models are provided in **Supplementary Table 2**.

### Statistical analysis and model evaluation

Continuous variables were assessed for normality. Normally distributed variables were presented as mean ± standard deviation and compared using the independent-samples t test, whereas non-normally distributed variables were presented as median and interquartile range and compared using the Mann–Whitney U test. Categorical variables were presented as number and percentage and compared using the χ² test. Multicollinearity among the selected clinical variables was assessed using variance inflation factors (VIFs). Potential nonlinear associations with early-stage DR were evaluated using restricted cubic spline analyses, and pairwise interactions among the selected variables were assessed using interaction terms. The clinical model, UW-OCTA image-based model, and multimodal model were evaluated in the internal test set and external validation set. Model discrimination was assessed using receiver operating characteristic curve analysis, and the area under the curve was calculated. Pairwise differences between their correlated AUCs were assessed within each set using two-sided paired DeLong tests, with ΔAUCs, 95% confidence intervals, and P values reported. Calibration of the multimodal model was assessed separately in the internal test set and external validation set using calibration plots, calibration intercepts, and calibration slopes; overall probabilistic accuracy was evaluated using the Brier score. Sensitivity, specificity, accuracy, positive predictive value (PPV), negative predictive value (NPV) and F1 score were also calculated to evaluate classification performance. Two ophthalmologists who were not involved in establishing the reference standard independently reviewed the UW-OCTA images and clinical information for all participants in both sets. Their sensitivity and specificity were calculated against the reference standard and compared descriptively with those of the multimodal model. For logistic regression analysis, odds ratios and 95% confidence intervals were reported. All statistical tests were two-sided, and P<0.05 was considered statistically significant. Statistical analyses were performed using SPSS version 26.0, R4.6.1 and Python version 3.10.6.

### Interpretability analysis of the OCTA image-based model

Gradient-weighted class activation mapping (Grad-CAM) was applied to the UW-OCTA image-based model to visualize retinal regions contributing to model classification. Heatmaps were generated from the final convolutional layer and overlaid on the original UW-OCTA images. Representative heatmaps from participants with early-stage DR and those without DR were compared qualitatively to assess whether the model focused on clinically relevant retinal regions. For expert validation, two ophthalmologists reviewed all early-stage DR cases in the internal test and external validation cohorts, and spatial correspondence between clinically relevant OCTA abnormalities and Grad-CAM high-activation regions was assessed by consensus.

## Results

### Study population and baseline characteristics

A total of 1,676 participants with T2DM were included in this study, comprising 1,047 in the training set (early-stage DR, n = 354; NDR, n = 693), 453 in the internal test set (early-stage DR, n = 151; NDR, n = 302), and 176 in the external validation set (early-stage DR, n = 38; NDR, n = 138) (**Figure 1**). The baseline characteristics of the study population are shown in **Table 1**. The external validation set had a lower proportion of male participants than the overall population, training set, and internal test set (45.45% vs. 60.62%, 63.04%, and 61.15%) and a higher median age (68 vs. 57, 56, and 55 years). Glycated hemoglobin (HbA1c) was lower in the external validation set than in the other datasets (7.0% vs. 8.2%, 8.5%, and 8.6%). Patients in the training and internal test sets were recruited from an urban tertiary medical center, whereas those in the external validation set were recruited from a primary community healthcare setting. This design enabled the evaluation of model generalizability across different patient sources and clinical settings.

**Figure 1:**
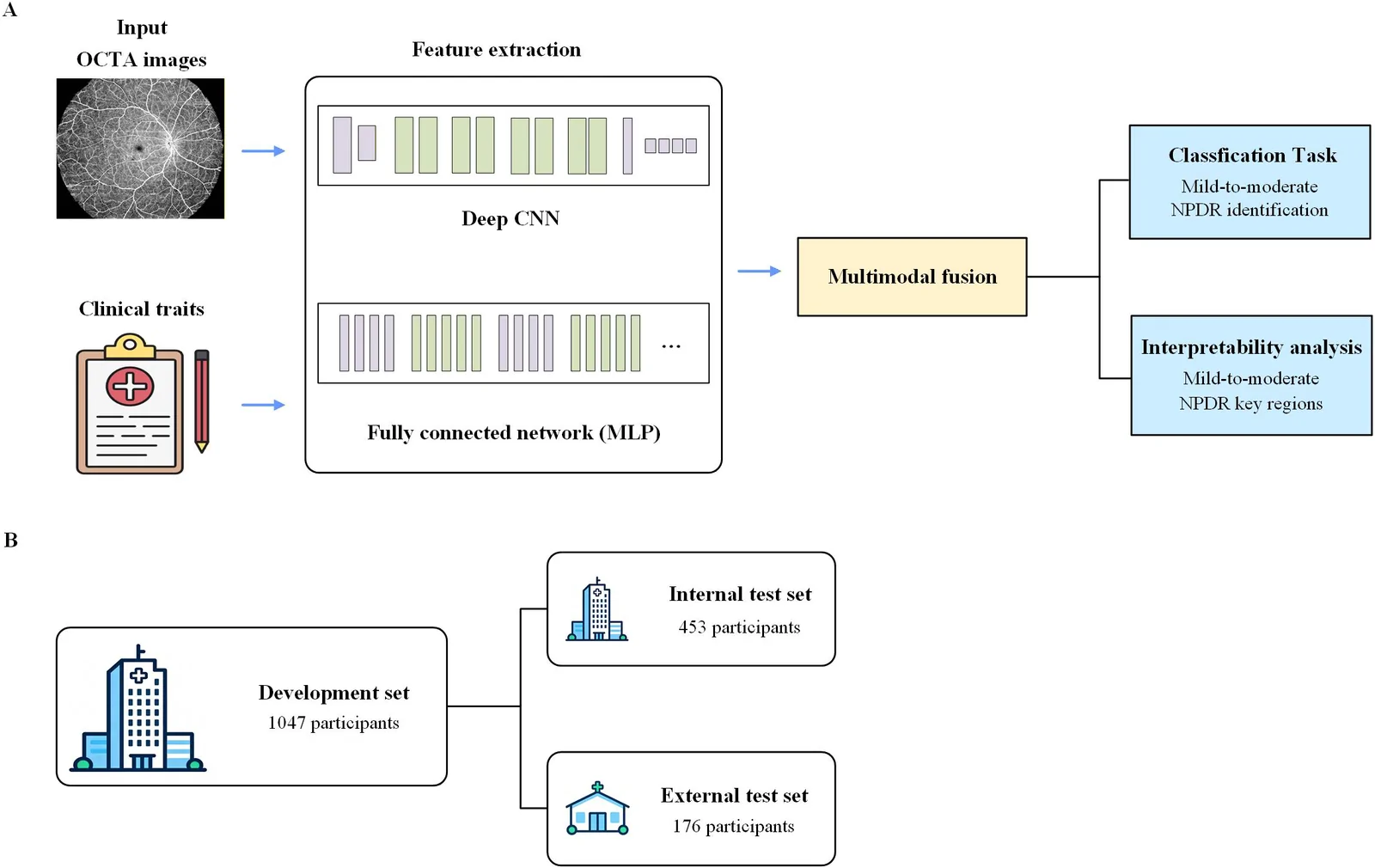
Multimodal deep learning workflow for detection of early-stage DR. **(A)** Model overview: UW-OCTA images and clinical features were input into a multimodal deep learning model. **(B)** Study cohorts: Patients were divided into development, internal test, and external validation sets for model evaluation.

**Table 1:** Baseline characteristics of study participants in the training, internal test, and external validation sets.

Characteristics	Total	Training data	Internal test data	External validation data
Number of participants	1676	1047	453	176
Hypertension, n(%)	727 (43.38%)	430 (41.07%)	185 (40.84%)	114 (64.77%)
Smoking History, n(%)	448 (26.73%)	290 (27.70%)	119 (26.27%)	39 (22.16%)
Alcohol Drinking History, n(%)	372 (22.20%)	227 (21.68%)	96 (21.19%)	49 (27.84%)
Male, n(%)	1016 (60.62%)	660 (63.04%)	277 (61.15%)	80 (45.45%)
Age, years	57 (48, 65)	56 (47, 63)	55 (47, 63)	68 (63, 72.5)
Diabetes duration, years	7 (2, 12)	6 (2, 12)	7 (2, 14)	7 (3, 14)
SBP, mmHg	135 (122, 149)	132 (120, 147)	134 (122, 149)	151 (139, 167)
DBP, mmHg	83 (76, 91)	83 (76, 91)	84 (75, 92)	83 (78, 87)
BMI, kg/m²	25.37 (23.31, 27.64)	25.34 (23.11, 27.47)	25.25 (23.23, 27.47)	26.16 (24.10, 28.12)
WBC, ×10^9^/L	6.15 (5.22, 7.37)	6.22 (5.17, 7.40)	6.03 (5.20, 7.22)	6.13 (5.40, 7.68)
Neutrophil, ×10^9^/L	3.50 (2.82, 4.37)	3.54 (2.81, 4.41)	3.41 (2.83, 4.34)	3.59 (2.84, 4.30)
Lymphocyte, ×10^9^/L	1.95 (1.56, 2.38)	1.94 (1.55, 2.38)	1.91 (1.51, 2.29)	2.12 (1.78, 2.63)
RBC, ×10¹²/L	4.85 (4.47, 5.20)	4.86 (4.49, 5.21)	4.90 (4.51, 5.22)	4.57 (4.30, 5.00)
Hb, g/L	149 (136, 160)	149 (136, 160)	149 (137, 160)	150 (139, 159)
PLT, ×10^9^/L	215 (183, 254)	218 (186, 255)	215 (183, 255)	207 (162, 249)
ALT, U/L	21.6 (16.0, 32.1)	21.2 (15.4, 32.6)	21.9 (16.1, 34.1)	22.4 (18.0, 28.0)
AST, U/L	19.9 (16.2, 26.2)	19.9 (16.1, 26.1)	19.8 (16.2, 27.4)	20.2 (17.0, 25.0)
TP, g/L	69.9 (65.6, 73.8)	70.1 (65.6, 73.9)	69.5 (65.4, 73.7)	/
ALB, g/L	42.5 (40.2, 45.2)	42.5 (40.2, 45.2)	42.7 (40.4, 45.1)	/
GLB, g/L	27.10 (24.20, 29.90)	27.20 (24.28, 30.03)	26.70 (24.00, 29.50)	/
BUN, mmol/L	5.58 (4.67, 6.63)	5.60 (4.67, 6.70)	5.69 (4.80, 6.56)	5.28 (4.26, 6.33)
UA, μmol/L	325.3 (266.1, 397.5)	325.7 (265.0, 398.7)	324.9 (268.2, 396.6)	/
FBG, mmol/L	8.20 (6.79, 10.38)	8.22 (6.74, 10.46)	8.29 (6.76, 10.56)	8.12 (6.92, 10.21)
TC, mmol/L	4.87 (4.01, 5.72)	4.79 (3.93, 5.60)	4.84 (3.99, 5.78)	5.45 (4.42, 6.22)
TG, mmol/L	1.75 (1.17, 2.82)	1.68 (1.15, 2.56)	1.76 (1.16, 2.93)	2.13 (1.38, 3.28)
LDL-C, mmol/L	3.02 (2.31, 3.71)	2.98 (2.24, 3.65)	3.00 (2.34, 3.77)	3.34 (2.65, 3.91)
HDL-C, mmol/L	1.13 (0.98, 1.37)	1.10 (0.96, 1.30)	1.10 (0.95, 1.29)	1.55 (1.33, 1.73)
HbA1c (%)	8.2 (7.0, 9.6)	8.5 (7.2, 9.8)	8.6 (7.4, 10.0)	7.0 (6.5, 7.9)
FINS, uIU/mL	10.51 (6.73, 15.82)	10.61 (6.79, 15.88)	10.37 (6.58, 15.65)	/
FCP, ng/mL	1.99 (1.40, 2.76)	2.02 (1.40, 2.81)	1.91 (1.37, 2.65)	/

Data are presented as n (%) for categorical variables and median (interquartile range) for continuous variables. The training and internal test sets were derived from an urban tertiary medical center, whereas the external validation set was derived from a primary community healthcare setting.

T2DM, type 2 diabetes mellitus; SBP, systolic blood pressure; DBP, diastolic blood pressure; BMI, body mass index; WBC, white blood cell count; RBC, red blood cell count; Hb, hemoglobin; PLT, platelet count; ALT, alanine aminotransferase; AST, aspartate aminotransferase; TP, total protein; ALB, albumin; GLB, globulin; BUN, blood urea nitrogen; UA, uric acid; FBG, fasting blood glucose; TC, total cholesterol; TG, triglyceride; LDL-C, low-density lipoprotein cholesterol; HDL-C, high-density lipoprotein cholesterol; HbA1c, glycated hemoglobin; FINS, fasting insulin; FCP, fasting C-peptide.

### Clinical feature selection and independent association analysis

In the training set, 354 participants had early-stage DR, whereas 693 participants had non-DR. Comparisons of clinical characteristics between the two groups showed significant differences in hypertension, duration of diabetes, systolic blood pressure (SBP), blood urea nitrogen (BUN), total cholesterol (TC), low-density lipoprotein cholesterol (LDL-C), and HbA1c (P<0.05) (**Supplementary Table 3**). SHAP-based feature importance analysis of the XGBoost model indicated that diabetes duration contributed most to the model output, followed by HbA1c, BUN, LDL-C, SBP, TC and hypertension (**Figure 2A**). Binary logistic regression analysis showed that diabetes duration (OR = 1.087, 95% CI: 1.066–1.109, P < 0.01), HbA1c (OR = 1.213, 95% CI: 1.121–1.313, P < 0.01), LDL-C (OR = 1.169, 95% CI: 1.009–1.353, P < 0.01), and SBP (OR = 1.015, 95% CI: 1.008–1.022, P < 0.01) were independently associated with early-stage DR (**Figure 2B**). No substantial multicollinearity was observed among the four selected variables (**Supplementary Table 4**). RCS analyses showed no significant nonlinearity, and no significant pairwise interactions were detected (all P for interaction > 0.05) (**Supplementary Figure 2**; **Supplementary Table 5**). These findings indicate that the four clinical variables hold significant detection value for early-stage DR, and were consequently used to develop a multimodal model to enable accurate early identification of DR.

**Figure 2:**
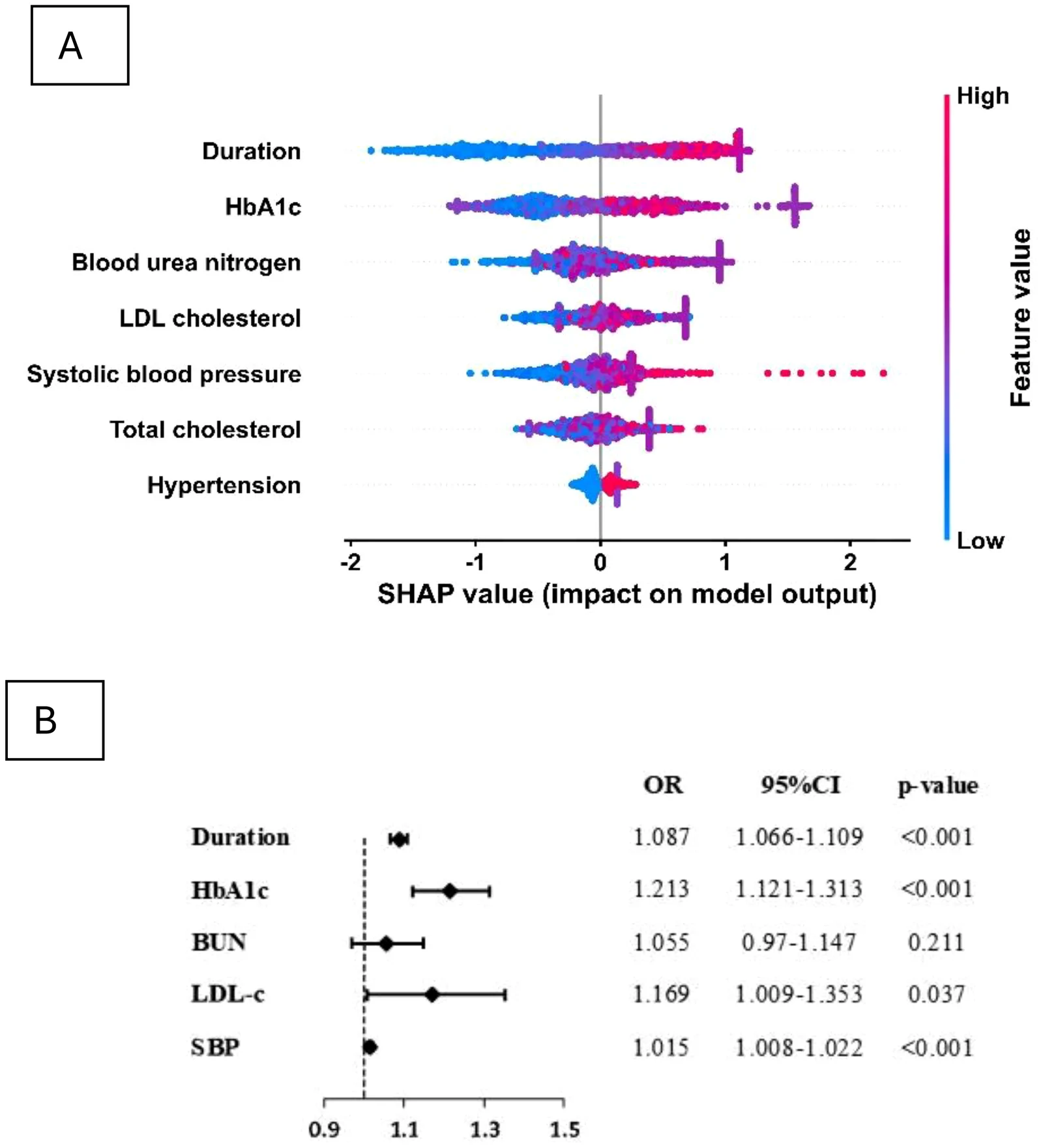
SHAP-based feature importance analysis and binary logistic regression of candidate clinical variables. **(A)** SHAP summary plot showing the relative importance of candidate clinical features; **(B)** Forest plot of odds ratios from the binary logistic regression model.

### Comparison of different UW-OCTA image layers for early DR identification

We compared the performance of four automatically derived 26 × 21 mm UW-OCTA layers, including RL, SCP, ICP and DCP for early-stage DR identification. In the internal test set, RL images showed the highest discriminative value, with an AUC of 0.906 (95%CI: 0.870-0.942), followed by the SCP, ICP and DCP, with AUC of 0.885 (95%CI: 0.846-0.925), 0.865 (95%CI: 0.823-0.907) and 0.851 (95%CI: 0.807-0.895), respectively (**Figure 3A**). This trend was further confirmed in the external validation set, in which RL images continued to demonstrate the highest discriminative ability, with an AUC of 0.828 (95%CI: 0.725-0.931), whereas the AUC of the SCP, ICP and DCP were 0.807 (95%CI: 0.700-0.915), 0.789 (95%CI: 0.679-0.900) and 0.779 (95%CI: 0.667-0.892), respectively (**Figure 3B**). These findings suggest that, among 26 × 21 mm UW-OCTA images, RL images provide the most stable discriminative performance for early-stage DR. Therefore, RL images were selected as the UW-OCTA imaging input for the subsequent multimodal model.

**Figure 3:**
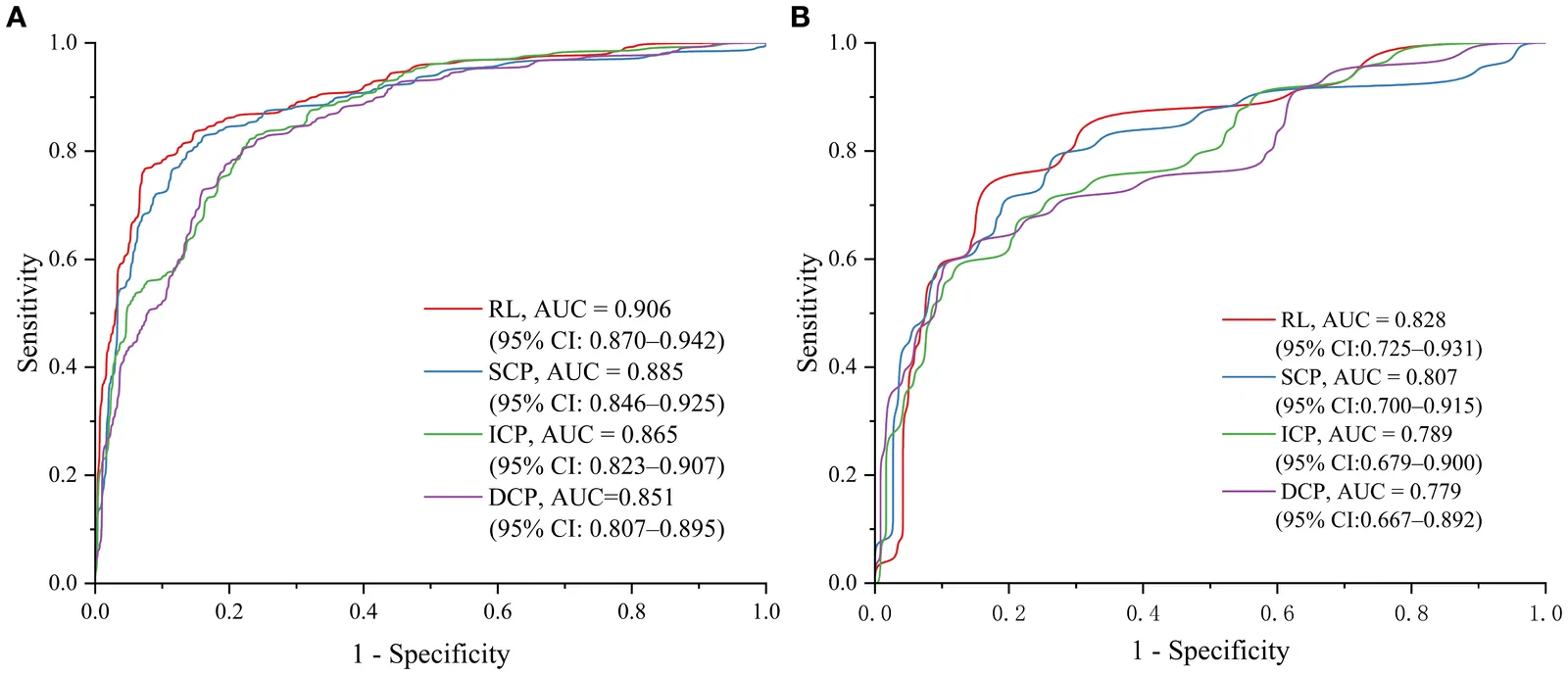
Performance of different OCTA retinal layers in identifying early-stage DR. **(A)** ROC curves showing the performance of different OCTA layers in the internal test set; **(B)** ROC curves showing the performance of different OCTA layers in the external validation set. RL, retinal layer; SCP, superficial capillary plexus; ICP, intermediate capillary plexus; DCP, deep capillary plexus.

### Comparative performance of the clinical model, UW-OCTA image model, and multimodal model for identifying early-stage DR

Based on the previously identified clinically relevant factors, a clinical model was constructed. In parallel, an UW-OCTA image-based DL model was developed using UW-OCTA images of the RL, which showed the best discriminative performance. Clinical features and UW-OCTA image features were further integrated to construct a multimodal combined model (**Figure 1A**). The discriminative performance of the three models was subsequently evaluated in both the internal test set and external validation set. In the internal test set, the AUCs of the multimodal combined model, UW-OCTA image-based model and clinical model were 0.921 (95%CI: 0.887-0.954), 0.906 (95%CI: 0.870-0.942) and 0.741 (95%CI: 0.690-0.792), respectively (**Figure 4A**; **Supplementary Table 6**). Among these models, the multimodal combined model achieved the highest AUC, and paired DeLong tests confirmed that its AUC was significantly higher than those of both the clinical model and the UW-OCTA image-based model (**Supplementary Table 7**), suggesting that integration of clinical features and UW-OCTA image features could further improve the identification of early-stage DR. In the external validation set, the AUCs of the multimodal combined model, UW-OCTA image-based model, and clinical model were 0.849 (95%CI: 0.760-0.953), 0.828 (95%CI: 0.725-0.931) and 0.744 (95%CI: 0.644-0.843), respectively (**Figure 4B**; **Supplementary Table 6**). Consistent with the internal test results, the multimodal combined model continued to show the best discriminative ability in the external validation set (**Supplementary Table 7**). The multimodal model also demonstrated generally acceptable calibration in both the internal test and external validation sets (**Supplementary Figure 1**; **Supplementary Table 8**). Furthermore, in both cohorts, the sensitivity and specificity of the multimodal model were broadly similar to those of the two ophthalmologists (**Supplementary Table 9**). Taken together, these findings suggest that clinical information and UW-OCTA imaging information provide complementary value and that their integration may improve the identification of early-stage DR in patients with T2DM.

**Figure 4:**
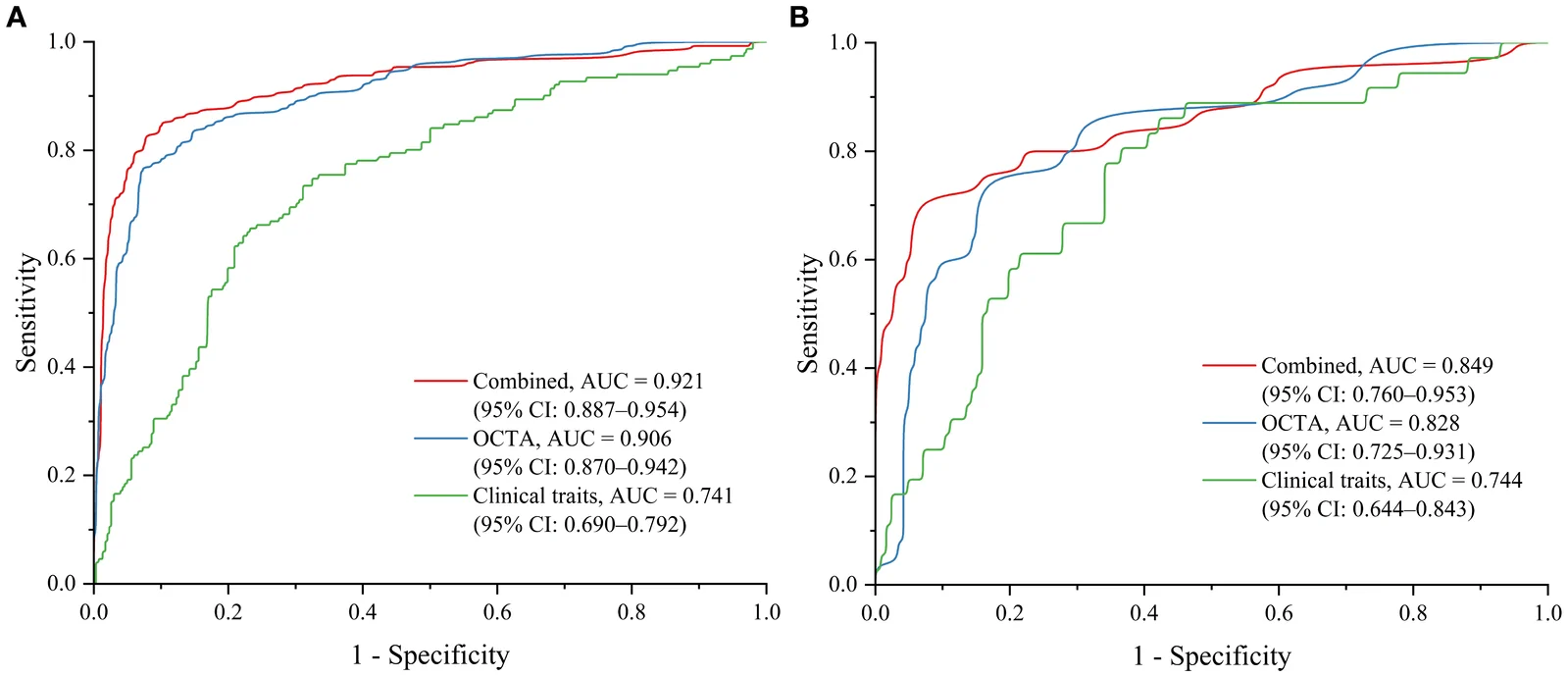
Comparative performance of clinical, OCTA image, and multimodal models for early-stage DR identification. **(A)** ROC curves for clinical factors, OCTA images, and their combination in the internal test set; **(B)** ROC curves for clinical factors, OCTA images, and their combination in the external validation set.

### Grad-CAM-based model interpretability analysis

To further investigate the discriminative basis of the UW-OCTA image-based DL model, Grad-CAM was applied to visualize the regions attended by the model. The results showed that, in eyes with early-stage DR, the heatmaps exhibited distinct focal high-activation regions. The model’s feature activation regions are predominantly localized to the macula, with additional extensive distribution across the peripheral retina outside the macular area. Expert-assessed spatial correspondence between clinically relevant OCTA abnormalities and Grad-CAM high-activation regions was observed in 156 of 189 early-stage DR cases (82.5%). Notably, some visible microvascular abnormalities showed good spatial correspondence with the model’s high-activation regions. These findings suggest that, during the identification of early-stage DR, the UW-OCTA image-based model not only utilized microvascular information from the posterior pole and macular region, but also captured peripheral retinal features provided by UW-OCTA imaging, thereby offering a degree of interpretability for the model’s discriminative performance (**Figure 5**).

**Figure 5:**
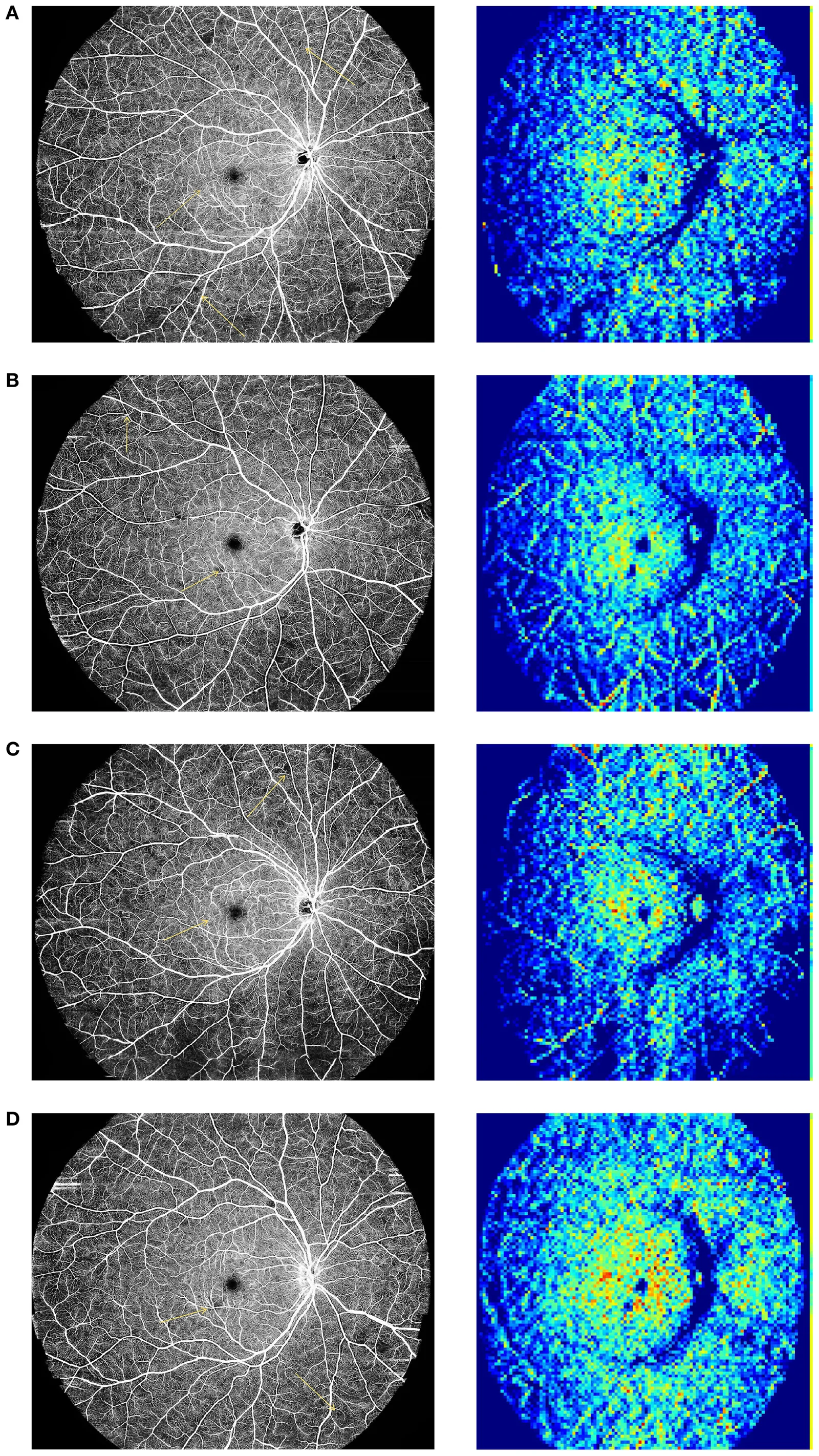
Grad-CAM visualization of model attention in early-stage DR identification. **(A–D)** Representative examples from four eyes with early-stage DR. In each pair, the left panel shows the original UW-OCTA image, and the right panel shows the corresponding Grad-CAM heatmap. Clinically relevant OCTA abnormalities identified by ophthalmologists are indicated by arrows for visual comparison with Grad-CAM high-activation regions. Warmer colors indicate regions that contributed more strongly to the model’s classification decision. The high-activation areas were mainly located in the macular, with additional activation extending to extramacular and peripheral retinal areas.

## Discussion

This study systematically evaluated the value of different automatically segmented UW-OCTA layers and routine clinical factors and further developed an image–clinical multimodal DL model for early-stage DR identification. First, among the different UW-OCTA layers, retinal layer images demonstrated the highest discriminative performance. Second, diabetes duration, HbA1c, LDL-C and SBP were identified as clinical factors independently associated with early-stage DR. Based on these imaging and clinical feature selection results, the multimodal model achieved the highest discriminative performance in both the internal test set and the external validation set, with AUCs of 0.921 (95%CI: 0.887-0.954) and 0.849 (95%CI: 0.760-0.953), respectively. These values were higher than those of the UW-OCTA image-only model (AUCs of 0.906 (95%CI: 0.870-0.942) and 0.828 (95%CI: 0.725-0.931), respectively) and the clinical data-only model (AUCs of 0.741 (95%CI: 0.690-0.792) and 0.744 (95%CI: 0.644-0.843), respectively). These findings suggest that integrating UW-OCTA-derived imaging features with clinical features may provide complementary information and enable a more comprehensive identification of early-stage DR in patients with T2DM. Further Grad-CAM visualization showed that the discriminative regions of the model were mainly located in the macular region and peripheral retina region within the UW-OCTA field, suggesting that the multimodal model may integrate retinal microvascular abnormalities from both the posterior pole and a wider retinal field. Overall, combining non-invasive UW-OCTA imaging with routine clinical indicators may provide an objective, accessible, and potentially translatable strategy for early-stage DR identification in patients with T2DM.

Identification of early-stage DR is essential for timely intervention and the prevention of vision-threatening complications in patients with diabetes. Recent studies have shown the potential of AI in the identification of DR (16). Dai et al. developed DeepDR, a DL system that facilitates automated DR assessment and enables detection of DR from its early stages (17). However, most existing AI-based DR detection approaches rely on fundus photography (18), which provides limited information on early retinal microvascular impairment. UW-OCTA can non-invasively visualize retinal microvascular alterations and owing to its wider imaging field compared with conventional macula-centered small-field OCTA, may further capture perfusion abnormalities in extramacular mid-peripheral regions, thereby providing additional imaging information for early-stage DR identification (19, 20). Nevertheless, AI studies using UW-OCTA for DR detection remain scarce and have mainly focused on public benchmark datasets or algorithm validation (21), with limited attention to the early identification of early-stage DR. In this study, we developed a deep learning model based on UW-OCTA images, providing a reference for the early detection of DR.

OCTA systems can automatically segment retinal vascular images into different slabs (22). However, no consensus has been reached regarding the optimal OCTA imaging slab for detecting DR. In the present study, we further compared the ability of different UW-OCTA slabs to identify early-stage DR in both the internal test set and the external validation set. Our study found that the RL exhibited the highest discriminative performance in the internal test set, with an AUC of 0.906 (95% CI: 0.870–0.942), compared with 0.885 (95% CI: 0.846–0.925) for the SCP, 0.865 (95% CI: 0.823–0.907) for the ICP, and 0.851 (95% CI: 0.807–0.895) for the DCP. This finding was further confirmed in the external validation set, with AUCs of 0.828 (95% CI: 0.725–0.931), 0.807 (95% CI: 0.700–0.915), 0.789 (95% CI: 0.679–0.900), and 0.779 (95% CI: 0.667–0.892), respectively. The higher discriminative performance of full-thickness retinal images may be attributed to their ability to comprehensively represent blood flow features from both the superficial and deep retinal vascular plexuses, thereby capturing early-stage DR-related microvascular abnormalities more completely than single-layer images (17, 23). In addition, because the full-thickness retinal slab aggregates information from multiple vascular layers, it inherently provides a broader range of vascular features than any individual plexus, which may also contribute to its superior discriminative performance. In line with our findings, Ebrahimi et al. compared single-layer OCTA inputs with multilayer fusion strategies for deep learning-based DR classification and demonstrated that multilayer fusion further improved classification performance (24), suggesting that integrated multilayer OCTA information may capture DR-related microvascular impairment more comprehensively than single-slab images. Therefore, the full-thickness retinal image was selected as the OCTA input for the subsequent multimodal model, providing a basis for the early identification of DR.

In addition to ocular imaging features, routine clinical indicators may provide important complementary information for the identification of early-stage DR. In the internal training cohort of this study, diabetes duration (OR = 1.087, 95% CI: 1.066–1.109), HbA1c (OR = 1.213, 95% CI: 1.121–1.313), LDL-C (OR = 1.169, 95% CI: 1.009–1.353) and SBP (OR = 1.015, 95% CI: 1.008–1.022) were all independently associated with early-stage DR. No evidence of multicollinearity was observed among these variables, and restricted cubic spline analyses showed no significant deviation from linearity in their associations with early-stage DR. Previous epidemiological studies have also shown that diabetes duration, glycemic control, blood pressure, and dyslipidemia are important clinical factors associated with the development, progression, and risk stratification of DR (12, 25, 26). These indicators reflect long-term diabetic exposure, chronic hyperglycemia, dyslipidemia, and hemodynamic burden, respectively, and may be involved in the development of early-stage DR through processes such as retinal microvascular endothelial injury, blood–retinal barrier disruption, and local inflammation (27, 28). Therefore, incorporating these clinical indicators into the multimodal model may provide additional systemic metabolic information beyond UW-OCTA imaging features alone, thereby improving the comprehensiveness and clinical interpretability of early-stage DR identification.

In this study, the combination of UW-OCTA images and key clinical indicators demonstrated superior performance compared with either modality alone. Specifically, the multimodal model achieved AUCs of 0.921 (95%CI: 0.887-0.954) and 0.849 (95%CI: 0.760-0.953) in the internal test set and external validation set, respectively, compared with 0.906 (95%CI: 0.870-0.942) and 0.828 (95%CI: 0.725-0.931) for the UW-OCTA image-only model and 0.741 (95%CI: 0.690-0.792) and 0.744 (95%CI: 0.644-0.843) for the clinical indicator-only model. Notably, the external community-based cohort was older and had a distinct demographic and clinical profile, including a lower proportion of male participants, higher SBP and LDL-C levels, and lower HbA1c levels than the tertiary-care cohorts. The lower prevalence of early-stage DR in the community cohort (21.6% vs. 33.7% in the tertiary-care cohort) may also reflect differences in case mix between community-based and hospitalized populations. Despite these population differences, the multimodal model maintained good discriminative performance in the external cohort, supporting its potential generalizability across different clinical settings. The model also demonstrated acceptable calibration, with sensitivity and specificity comparable to those of two experienced ophthalmologists.

These findings suggest that UW-OCTA images and clinical indicators provide complementary information and may contribute to the identification of early-stage DR. Sandhu et al. reported that adding clinical and demographic variables to OCT-derived and OCTA-derived imaging biomarkers further improved the diagnostic performance for DR (29). Similarly, Li et al. found that OCTA parameters combined with clinical data were useful for classifying DR, referable DR, and vision-threatening DR (30). In addition, DeepDR Plus integrated retinal images with clinical data and showed superior performance in predicting DR progression, further supporting the value of combining retinal imaging findings with systemic risk factors (17).

Compared with extensively studied fundus photograph-based DL systems, OCTA provides noninvasive, flow-based, and layer-resolved visualization of the retinal microvasculature. Importantly, unlike previous OCT/OCTA-based multimodal studies that primarily combined predefined quantitative imaging biomarkers with clinical variables (29, 30), our framework learned deep spatial features directly from 26 × 21-mm UW-OCTA images and fused them at the feature level with four routine clinical factors. This design retained widefield spatial microvascular information and focused specifically on distinguishing early-stage DR from NDR. Taken together, these findings support the value of integrating UW-OCTA images with routine clinical factors for identifying early-stage DR.

The improved performance of the multimodal model over single-modality models may be explained by the complementary information provided by UW-OCTA imaging features and clinical indicators in characterizing early-stage DR. DR not only reflects local retinal microvascular injury but also represents the cumulative effects of diabetes-related systemic metabolic abnormalities on the retinal microcirculation (31). UW-OCTA images can directly visualize retinal microvascular morphology and perfusion abnormalities, providing imaging evidence of local ocular changes in early DR (19, 32). In contrast, clinical indicators such as diabetes duration, HbA1c, LDL-C, and SBP reflect long-term diabetes exposure, chronic glycemic control, lipid metabolic status, and hemodynamic burden, respectively (5). Therefore, integrating UW-OCTA images with key clinical indicators may simultaneously capture local retinal microvascular abnormalities and systemic metabolic risk, providing a clinically meaningful approach for the identification of early-stage DR.

The Grad-CAM heatmaps in this study showed that the model activation was not confined to the macular region but also extended to extramacular peripheral retinal regions within the UW-OCTA field. Notably, these high-activation regions frequently showed spatial correspondence with clinically relevant OCTA abnormalities identified by ophthalmologists. The macular region is an important site for observing early microcirculatory impairment in DR. Previous deep learning studies based on OCTA images have also shown that model discrimination primarily focuses on vascular enlargement around the foveal avascular zone (FAZ) and adjacent areas of capillary dropout, suggesting that macular microvascular abnormalities are important imaging features for automated DR identification (33). However, DR-related microvascular abnormalities are not limited to the macular region; extramacular retinal areas may also exhibit nonperfusion and predominantly peripheral lesions (34). Previous studies have demonstrated that such peripheral lesions are associated with DR severity and can independently predict future DR progression, indicating that they may provide additional early risk information beyond macula-centered assessment (35, 36). Moreover, peripheral retinal ischemia has been associated with diabetic macular edema and central macular ischemia (37, 38), suggesting that extramacular microvascular damage may contribute to subsequent macular involvement. Therefore, assessment of extramacular regions within the UW-OCTA field may provide additional imaging evidence for DR identification. The visualization results of this study further support the incremental value of the wider field information provided by UW-OCTA and suggest that extramacular microvascular abnormalities may serve as important imaging clues for assisting early-stage DR identification, although Grad-CAM reflects model attention rather than a definitive biological explanation.

Several limitations should also be acknowledged. First, high-quality UW-OCTA images are required for model application; therefore, patients with small pupils, severe cataract, media opacity, poor fixation, or motion artifacts may not be suitable for this approach. Moreover, UW-OCTA requires specialized equipment and trained operators, which may restrict its accessibility, particularly in primary-care and resource-limited settings. Furthermore, the exclusion of poor-quality images may have introduced selection bias by preferentially retaining participants who were easier to image, thereby potentially yielding optimistic estimates of model performance under routine clinical conditions. Second, all participants were recruited in China and further large-scale, multiethnic, multicenter external validation is warranted to confirm the robustness and generalizability of the model across different populations and healthcare settings. Third, given the cross-sectional design, the model identifies existing early-stage DR rather than predicting future DR onset or progression. Prospective longitudinal studies are needed to evaluate its potential for predicting DR. Fourth, the tertiary-care development cohort may not fully represent community populations, potentially introducing spectrum bias. Although external validation in a community-based cohort partly addressed model transportability, residual domain shift due to differences in participant characteristics and disease prevalence cannot be excluded. Finally, prospective implementation studies are needed to confirm model calibration and real-world clinical utility across populations, imaging devices, and healthcare settings.

In conclusion, integrating UW-OCTA imaging with routine clinical factors may improve the identification of early-stage DR in patients with T2DM. By incorporating retinal microvascular information from the macular and extramacular peripheral regions together with systemic metabolic risk profiles, this strategy may provide a clinically accessible basis for early DR risk assessment and timely intervention in patients with T2DM.

## Data Availability

The original contributions presented in the study are included in the article/**Supplementary Material**. Further inquiries can be directed to the corresponding author.

## References

[1] Cheung N Mitchell P Wong TY . Diabetic retinopathy. Lancet. (2010) 376:124–36. doi: 10.1016/s0140-6736(09)62124-3 20580421

[2] Teo ZL Tham YC Yu M Chee ML Rim TH Cheung N . Global prevalence of diabetic retinopathy and projection of burden through 2045: Systematic review and meta-analysis. Ophthalmology. (2021) 128:1580–91. doi: 10.1016/j.ophtha.2021.04.027 33940045

[3] Hou X Wang L Zhu D Guo L Weng J Zhang M . Prevalence of diabetic retinopathy and vision-threatening diabetic retinopathy in adults with diabetes in China. Nat Commun. (2023) 14:4296. doi: 10.1038/s41467-023-39864-w 37463878 PMC10354077

[4] Kropp M Golubnitschaja O Mazurakova A Koklesova L Sargheini N Vo TKS . Diabetic retinopathy as the leading cause of blindness and early predictor of cascading complications-risks and mitigation. EPMA J. (2023) 14:21–42. doi: 10.1007/s13167-023-00314-8 36866156 PMC9971534

[5] Donaghue KC Liew G . Measuring outcomes of diabetic retinopathy screening: What is important? Diabetes Care. (2024) 47:930–2. doi: 10.2337/dci24-0021 38768335

[6] Lu D Jiang J Zhao Y Anwar MS Ahmad PN Alhefdi M . DRD-MoE: Dominant-residual decomposed multi-router mixture-of-experts for blood glucose prediction. IEEE Trans Consum Electron. (2026). doi: 10.1109/tce.2026.371148025079929

[7] Lin Y Wang H Jiang J . KRC-APM: Key region cutting and artificial prior model for breast cancer recognition in ultrasound images. Expert Syst Appl. (2024) 257:125092. doi: 10.1016/j.eswa.2024.12509242574925

[8] Djoumessi K Huang Z Kühlewein L Rickmann A Simon N Koch LM . An inherently interpretable AI model improves screening speed and accuracy for early diabetic retinopathy. PloS Digit Health. (2025) 4:e0000831. doi: 10.1371/journal.pdig.0000831 40354306 PMC12068651

[9] Ran AR Ding JL Tang Z Lam C Nguyen TX Zhou J . Real-world prospective validation and economic evaluation of deep learning- based diabetic retinopathy detection from fundus photographs: A systematic review and meta-analysis. Diabetes Care. (2026) 49:510–25. doi: 10.2337/dc25-1493 41259706

[10] Daley JR Wang X Ngo N Khoo CL Heydon P Liew G . Development and validation of a deep learning system for the provision of a district-wide diabetes retinal screening service. Clin Exp Ophthalmol. (2025) 53:803–12. doi: 10.1111/ceo.14560 40491217 PMC12516661

[11] Jia Y Hormel TT Hwang TS Wu AL Liang GB Guo Y . Widefield OCT angiography. Prog Retin Eye Res. (2025) 107:101378. doi: 10.1016/j.preteyeres.2025.101378 40517946 PMC12342419

[12] Stratton IM Kohner EM Aldington SJ Turner RC Holman RR Manley SE . UKPDS 50: Risk factors for incidence and progression of retinopathy in type II diabetes over 6 years from diagnosis. Diabetologia. (2001) 44:156–63. doi: 10.1007/s001250051594 11270671

[13] Early Treatment Diabetic Retinopathy Study Research Group . Grading diabetic retinopathy from stereoscopic color fundus photographs--an extension of the modified Airlie House classification. ETDRS report number 10. Ophthalmology. (1991) 98:786–806. doi: 10.1016/S0161-6420(13)38012-9 2062513

[14] American Diabetes Association Professional Practice Committee for Diabetes 2 . Diagnosis and classification of diabetes: Standards of care in diabetes-2026. Diabetes Care. (2026) 49:S27–49. doi: 10.2337/dc26-s002 41358893 PMC12690183

[15] Hwang TS Zhang M Bhavsar K Zhang X Campbell JP Lin P . Visualization of 3 distinct retinal plexuses by projection-resolved optical coherence tomography angiography in diabetic retinopathy. JAMA Ophthalmol. (2016) 134:1411–9. doi: 10.1001/jamaophthalmol.2016.4272 27812696 PMC5805384

[16] Vujosevic S Aldington SJ Silva P Hernández C Scanlon P Peto T . Screening for diabetic retinopathy: New perspectives and challenges. Lancet Diabetes Endo. (2020) 8:337–47. doi: 10.1016/s2213-8587(19)30411-5 32113513

[17] Dai L Wu L Li H Cai C Wu Q Kong H . A deep learning system for detecting diabetic retinopathy across the disease spectrum. Nat Commun. (2021) 12:3242. doi: 10.1038/s41467-021-23458-5 34050158 PMC8163820

[18] Grzybowski A Jin K Zhou J Pan X Wang M Ye J . Retina fundus photograph-based artificial intelligence algorithms in medicine: A systematic review. Ophthalmol Ther. (2024) 13:2125–49. doi: 10.1007/s40123-024-00981-4 38913289 PMC11246322

[19] Xu F Li Z Gao Y Yang X Huang Z Li Z . Retinal microvascular signs in pre- and early-stage diabetic retinopathy detected using wide-field swept-source optical coherence tomographic angiography. J Clin Med. (2022) 11:4332. doi: 10.3390/jcm11154332 35893423 PMC9329884

[20] Le Boité H Bonnin S Gallardo M Lamard M Couturier A Quellec G . Deep learning for retinal non-perfusion and foveal avascular zone analysis in wide-field OCTA in diabetic retinopathy. Sci Rep. (2025) 15:30225. doi: 10.1038/s41598-025-15712-3 40826271 PMC12361365

[21] Qian B Chen H Wang X Guan Z Li T Jin Y . DRAC 2022: A public benchmark for diabetic retinopathy analysis on ultra-wide optical coherence tomography angiography images. Patterns (N Y). (2024) 5:100929. doi: 10.2139/ssrn.4593632 PMC10935505 38487802

[22] Garg I Uwakwe C Le R Lu ES Cui Y Wai KM . Nonperfusion area and other vascular metrics by wider field swept-source OCT angiography as biomarkers of diabetic retinopathy severity. Ophthalmol Sci. (2022) 2:100144. doi: 10.1016/j.xops.2022.100144 35647573 PMC9137369

[23] Lavia C Couturier A Erginay A Dupas B Tadayoni R Gaudric A . Reduced vessel density in the superficial and deep plexuses in diabetic retinopathy is associated with structural changes in corresponding retinal layers. PloS One. (2019) 14:e0219164. doi: 10.1371/journal.pone.0219164 31318880 PMC6638849

[24] Ebrahimi B Le D Abtahi M Dadzie AK Lim JI Chan RVP . Optimizing the OCTA layer fusion option for deep learning classification of diabetic retinopathy. BioMed Opt Express. (2023) 14:4713–24. doi: 10.1364/BOE.495999 PMC10545199 37791267

[25] UK Prospective Diabetes Study (UKPDS) Group . Intensive blood-glucose control with sulphonylureas or insulin compared with conventional treatment and risk of complications in patients with type 2 diabetes (UKPDS 33). Lancet. (1998) 352:837–53. doi: 10.1016/S0140-6736(98)07019-6 9742976

[26] UK Prospective Diabetes Study Group . Tight blood pressure control and risk of macrovascular and microvascular complications in type 2 diabetes: UKPDS 38. BMJ. (1998) 317:703–13. doi: 10.1136/bmj.317.7160.703 PMC28659 9732337

[27] Rudraraju M Narayanan SP Somanath PR . Regulation of blood-retinal barrier cell-junctions in diabetic retinopathy. Pharmacol Res. (2020) 161:105115. doi: 10.1016/j.phrs.2020.105115 32750417 PMC7755666

[28] Bryl A Mrugacz M Falkowski M Zorena K . The effect of hyperlipidemia on the course of diabetic retinopathy-literature review. J Clin Med. (2022) 11:2761. doi: 10.3390/jcm11102761 35628887 PMC9146710

[29] Sandhu HS Elmogy M Taher Sharafeldeen A Elsharkawy M El-Adawy N Eltanboly A . Automated diagnosis of diabetic retinopathy using clinical biomarkers, optical coherence tomography, and optical coherence tomography angiography. Am J Ophthalmoll. (2020) 216:201–6. doi: 10.1016/j.ajo.2020.01.016 31982407

[30] Li X Wen X Shang X Liu J Zhang L Cui Y . Identification of diabetic retinopathy classification using machine learning algorithms on clinical data and optical coherence tomography angiography. Eye (Lond). (2024) 38:2813–21. doi: 10.1038/s41433-024-03173-3 38871934 PMC11427469

[31] Yumnamcha T Guerra M Singh LP Ibrahim AS . Metabolic dysregulation and neurovascular dysfunction in diabetic retinopathy. Antioxidants (Basel). (2020) 9:1244. doi: 10.3390/antiox9121244 33302369 PMC7762582

[32] Wang XN Cai X Li SW Li T Long D Wu Q . Wide-field swept-source OCTA in the assessment of retinal microvasculature in early-stage diabetic retinopathy. BMC Ophthalmol. (2022) 22:473. doi: 10.1186/s12886-022-02724-0 36474199 PMC9727974

[33] Waheed NK Rosen RB Jia Y Munk MR Huang D Fawzi A . Optical coherence tomography angiography in diabetic retinopathy. Prog Retin Eye Res. (2023) 97:101206. doi: 10.1016/j.preteyeres.2023.101206 37499857 PMC11268430

[34] Silva PS Liu D Glassman AR Aiello LP Grover S Kingsley RM . Assessment of fluorescein angiography nonperfusion in eyes with diabetic retinopathy using ultrawide field retinal imaging. Retina. (2022) 42:1302–10. doi: 10.1097/iae.0000000000003479 35344528 PMC9233028

[35] Marcus DM Silva PS Liu D Aiello LP Antoszyk A Elman M . Association of predominantly peripheral lesions on ultra-widefield imaging and the risk of diabetic retinopathy worsening over time. JAMA Ophthalmol. (2022) 140:946–54. doi: 10.1001/jamaophthalmol.2022.3131 35980608 PMC9389433

[36] Silva PS Cavallerano JD Haddad NM Kwak H Dyer KH Omar AF . Peripheral lesions identified on ultrawide field imaging predict increased risk of diabetic retinopathy progression over 4 years. Ophthalmology. (2015) 122:949–56. doi: 10.1016/j.ophtha.2015.01.008 25704318

[37] Wessel MM Nair N Aaker GD Ehrlich JR D'Amico DJ Kiss S . Peripheral retinal ischaemia, as evaluated by ultra-widefield fluorescein angiography, is associated with diabetic macular oedema. Br J Ophthalmol. (2012) 96:694–8. doi: 10.1136/bjophthalmol-2011-300774 22423055 PMC3329634

[38] Hein M Mehnert A Josephine F Athwal A Yu DY Balaratnasingam C . Predictors of peripheral retinal non-perfusion in clinically significant diabetic macular edema. J Clin Med. (2024) 14:52. doi: 10.3390/jcm14010052 39797135 PMC11722121

