# Supplementary material for: Integration of ultra-widefield optical coherence tomography angiography images and clinical indicators for identifying early-stage diabetic retinopathy in type 2 diabetes
Source: Front Endocrinol (Lausanne). 2026 Sep 24;17:1941613. doi: 10.3389/fendo.2026.1941613 (PMC13645700; doi:10.3389/fendo.2026.1941613)
Supplement: Supplementary file 1 [file DataSheet1.pdf]

Supplementary Table 1. Participant selection and reasons for exclusion in the tertiary-care and community cohorts

Reason for exclusion	Tertiary-care cohort, n	Community cohort, n
Participants initially assessed	1696	192
Age <18 years	19	1
Did not undergo UW-OCTA examination	52	3
Other ocular diseases or previous ocular treatment	62	5
Poor-quality UW-OCTA images	42	5
Missing key clinical variables	21	2
Participants included in the final analysis	1500	176

Supplementary Table 2. Implementation and training details of the OCTA image-based and multimodal deep learning models.

Item	Training details
Learning rate	1×10^{-4}
weight decay	1×10^{-4}
Batch size	16
Number of training epochs	50
Model-selection criteria	A validation subset within the training cohort.
Random seed	N/A
Hardware/software environment	Python 3.10.6, PyTorch 2.11.0, CUDA 12.8, and an NVIDIA GeForce RTX 5050 GPU
Number of model parameters	UW-OCTA image-only model: 11.18 million<br>Multimodal model: 11.34 million
Whether the backbone was fully fine-tuned or partially frozen	Fully fine-tuned
How class imbalance was addressed	Class-weighted cross-entropy
Whether multiple training runs were performed	No
Whether the final model was selected from a validation subset within the training cohort	Yes
Additional implementation details identifiable from the code	Images resized: 224 × 224<br>Random rotation: 30°<br>Random horizontal flipping

Supplementary table 3. Clinical characteristics of participants with and without early-stage DR in the training set.

	Early-stage DR	NDR	Z/ χ^2	P

Number of participants	354	693		
Hypertension, n(%)	166 (46.89%)	267 (38.53%)	-2.49	0.013
Smoking History, n(%)	95 (26.84%)	195 (28.14%)	-0.442	0.658
Alcohol Drinking History, n(%)	79 (22.32%)	148 (21.36%)	-0.394	0.694
Male, n(%)	222 (62.71%)	472 (68.11%)	-0.117	0.907
Age, years	55（48，62）	56（46，63）	-0.19	0.85
Diabetes duration, years	10（5，16）	5（1，10）	-8.607	<0.001
SBP, mmHg	137（123，150）	130（119，145）	-3.963	<0.001
DBP, mmHg	83（76，93）	83（76，91）	-0.072	0.942
BMI, kg/m^2	25.25（23.14，27.04）	25.39（23.06，27.72）	-1.383	0.167
WBC, $\times 10^9$/L	6.20（5.11，7.55）	6.22（5.19，7.32）	-0.835	0.403
Neutrophil, $\times 10^9$/L	3.56（2.87，4.58）	3.53（2.76，4.35）	-1.528	0.127
Lymphocyte, $\times 10^9$/L	1.89（1.51，2.33）	1.95（1.57，2.39）	-1.387	0.165
RBC, $\times 10^{12}$/L	4.87（4.52，5.19）	4.86（4.48，5.22）	-0.141	0.888
Hb, g/L	148（136，158）	149（136，161）	-1.387	0.165
PLT, $\times 10^9$/L	219（189，255）	217（185，255）	-0.446	0.656
ALT, U/L	18.9（13.8，26.6）	22.3（16.4，35.0）	-5.142	<0.001
AST, U/L	18.5（15.6，22.9）	20.8（16.5，27.5）	-4.714	<0.001
TP, g/L	70.2（65.0，73.9）	70.0（65.9，73.7）	-0.174	0.862
ALB, g/L	42.1（39.6，44.6）	42.7（40.5，45.5）	-2.844	0.004
GLB, g/L	28.30（24.52，30.70）	26.90（24.20，29.60）	-2.849	0.004
BUN, mmol/L	5.83（4.83，6.80）	5.45（4.60，6.68）	-2.377	0.017
UA, μmol/L	317.9（255.6，394.0）	328.8（268.7，400.9）	-1.879	0.06
FBG, mmol/L	8.44（6.74，10.96）	8.09（6.73，10.11）	-1.834	0.067
TC, mmol/L	4.93（4.07，5.77）	4.75（3.88，5.55）	-2.172	0.03
TG, mmol/L	1.64（1.11，2.65）	1.69（1.15，2.56）	-0.633	0.527
LDL-C, mmol/L	3.09（2.39，3.68）	2.91（2.17，3.62）	-2.111	0.035
HDL-C, mmol/L	1.10（0.96，1.30）	1.10（0.96，1.29）	-0.124	0.901
HbA1c (%)	8.8（7.7，10.0）	8.2（7.1，9.7）	-3.747	<0.001
FINS, uIU/mL	10.23（6.32，16.69）	10.76（7.03，15.53）	-0.444	0.657
FCP, ng/mL	1.88（1.24，2.53）	2.10（1.46，2.94）	-3.66	<0.001

Notes: Data are presented as n (%) for categorical variables and as median (interquartile range) for continuous variables, as appropriate. Between-group comparisons were performed using the chi-square test or Fisher’s exact test for categorical variables and the Mann–Whitney U test or Student’s t-test for continuous variables, as appropriate.

Abbreviations: DR, diabetic retinopathy; NDR, non-diabetic retinopathy; SBP, systolic blood pressure; DBP, diastolic blood pressure; BMI, body mass index; WBC, white blood cell count; RBC, red blood cell count; Hb, hemoglobin; PLT, platelet count; ALT, alanine aminotransferase; AST, aspartate aminotransferase; TP, total protein; ALB, albumin; GLB, globulin; BUN, blood urea nitrogen; UA, uric acid;

FBG, fasting blood glucose; TC, total cholesterol; TG, triglyceride; LDL-C, low-density lipoprotein cholesterol; HDL-C, high-density lipoprotein cholesterol; HbA1c, glycated hemoglobin; FINS, fasting insulin; FCP, fasting C-peptide.

Supplementary Table 4. Multicollinearity assessment among the four selected clinical features in the training set.

Variable	VIF	Tolerance
Diabetes duration	1.038	0.963
HbA1c	1.050	0.952
LDL-C	1.043	0.959
SBP	1.018	0.982

Abbreviations: VIF, variance inflation factor; SBP, systolic blood pressure; LDL-C, low-density lipoprotein cholesterol; HbA1c, glycated hemoglobin.

Supplementary Table 5. Pairwise interaction analyses among the selected clinical factors for diabetic retinopathy

Interaction term	P for interaction
Duration × SBP	0.354
Duration × LDL-C	0.368
Duration × HbA1c	0.073
SBP × LDL-C	0.574
SBP × HbA1c	0.61
LDL-C × HbA1c	0.247

Abbreviations: VIF, variance inflation factor; SBP, systolic blood pressure; LDL-C, low-density lipoprotein cholesterol; HbA1c, glycated hemoglobin.

Supplementary Table 6. Performance of the clinical-only, UW-OCTA image-only, and multimodal models in the internal test set and .

	**AUC (95% CI)**	**Sensitivity (95% CI)**	**Specificity (95% CI)**	**Accuracy (95% CI)**	**PPV (95% CI)**	**NPV (95% CI)**	**F1 (95% CI)**
**internal test set (n=453)**							
**Clinical-only**	0.741 (0.690–0.792)	0.517 (0.444, 0.596)	0.861 (0.817, 0.895)	0.746 (0.693, 0.795)	0.650 (0.548, 0.739)	0.781 (0.746, 0.816)	0.576 (0.491, 0.660)
**OCTA-only**	0.906 (0.870-0.942)	0.781 (0.709–0.840)	0.907 (0.869–0.935)	0.865 (0.831–0.894)	0.808 (0.737–0.864)	0.893 (0.853–0.922)	0.795 (0.744–0.840)
**Multimodal**	0.921 (0.887-0.954)	0.742 (0.667–0.805)	0.950 (0.920–0.970)	0.881 (0.848–0.907)	0.882 (0.814–0.927)	0.880 (0.841–0.911)	0.806 (0.754–0.854)
**external validation set (n=176)**							
**Clinical-only**	0.744 (0.644–0.843)	0.579 (0.421, 0.737)	0.833 (0.759, 0.888)	0.778 (0.686, 0.855)	0.489 (0.325, 0.645)	0.878 (0.826, 0.925)	0.530 (0.367, 0.688)

**OCTA-only**	0.828 (0.725, 0.931)	0.632 (0.473–0.766)	0.935 (0.881–0.965)	0.869 (0.812–0.911)	0.727 (0.558–0.849)	0.902 (0.842–0.941)	0.676 (0.536–0.791)
**Multimodal**	0.849 (0.760, 0.953)	0.763 (0.608–0.870)	0.928 (0.872–0.960)	0.892 (0.838–0.930)	0.744 (0.589–0.854)	0.934 (0.880–0.965)	0.753 (0.649–0.849)

AUC, area under the receiver operating characteristic curve; CI, confidence interval; PPV, positive predictive value; NPV, negative predictive value.

Supplementary Table 7. Pairwise comparisons of model AUCs in the internal test and external validation sets using the paired DeLong test.

**Model comparison**	**Internal test set ΔAUC（95% CI）**	**P value**	**External validation set ΔAUC（95% CI）**	**P value**
Combined vs clinical traits	0.179（0.119–0.239）	<0.001	0.105（0.035–0.175）	0.003
Combined vs OCTA	0.014（0.003–0.025）	0.045	0.021（0.002–0.051）	0.039
OCTA vs clinical traits	0.165（0.105–0.225）	<0.001	0.084（0.013–0.155）	0.021

ΔAUC was calculated as the AUC of the first model minus that of the second model. P values were obtained using two-sided paired DeLong tests.

Supplementary Table 8. Calibration performance of the multimodal model in the internal test and external validation sets.

**Cohort**	**Brier score**	**Calibration intercept**	**Calibration slope**
Internal test set	0.104	-0.063	0.933
External validation set	0.112	-0.243	0.879

Supplementary Table 9. Performance of the multimodal model and ophthalmologists for early-stage DR identification

**Cohort**	**Metric**	**Multimodal model**	**Ophthalmologist 1**	**Ophthalmologist 2**
Internal test	Sensitivity, (95% CI)	0.742 (0.667–0.805)	0.735 (0.660–0.799)	0.724 (0.648–0.789)
	Specificity, (95% CI)	0.950 (0.920–0.970)	0.990 (0.971–0.997)	0.993 (0.976–0.998)
External validation	Sensitivity, (95% CI)	0.763 (0.608–0.870)	0.737 (0.580–0.850)	0.710 (0.552–0.830)
	Specificity, (95% CI)	0.928 (0.872–0.960)	0.971 (0.928–0.989)	0.964 (0.918-0.985)

1

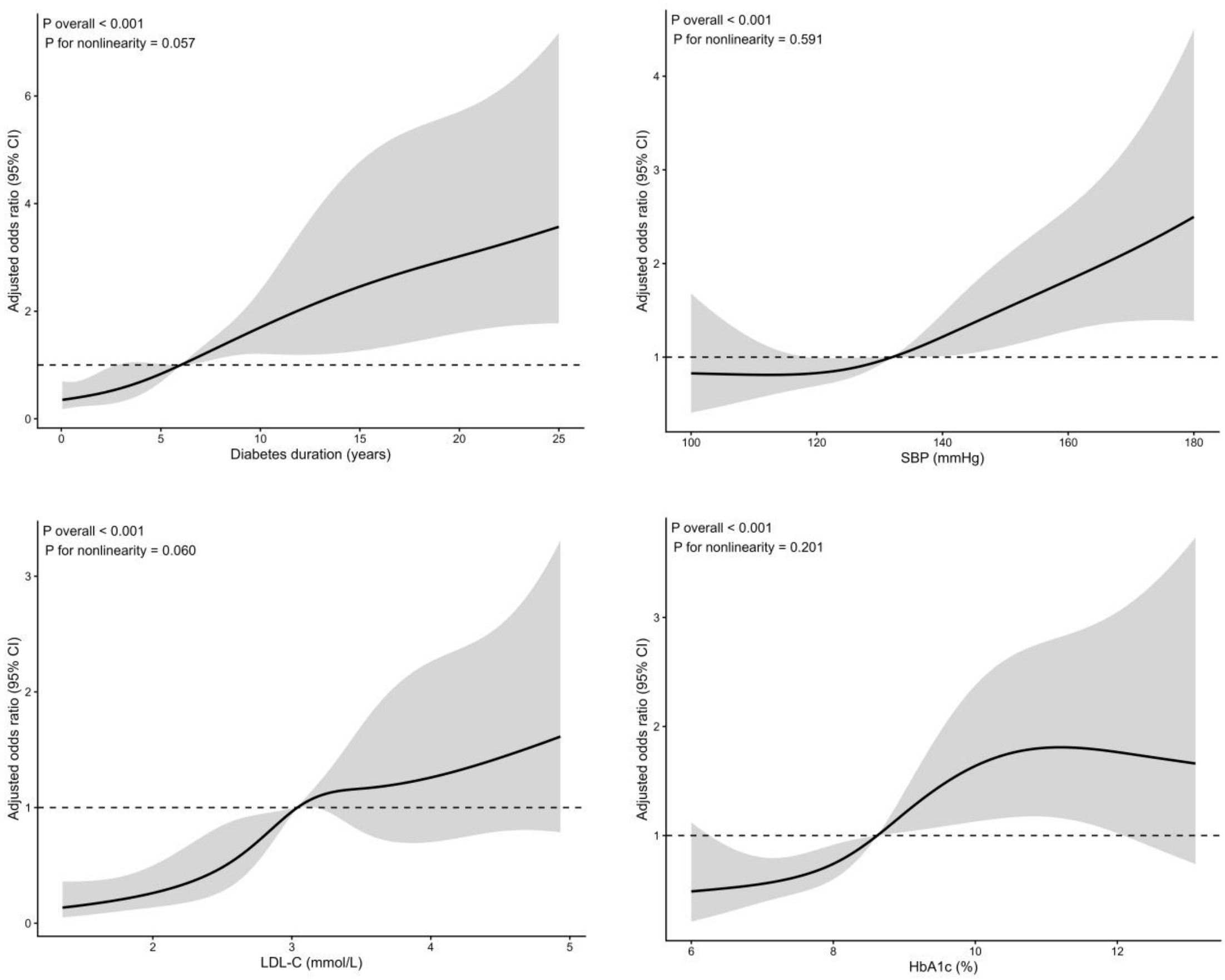

Supplementary Figure 1. Restricted cubic spline analyses of the associations between the four selected clinical features and early-stage diabetic retinopathy.

Notes: Restricted cubic spline analyses of the associations of (A) diabetes duration, (B) systolic blood pressure, (C) low-density lipoprotein cholesterol, and (D) glycated hemoglobin with early-stage diabetic retinopathy. The curves represent the estimated odds ratios, and the shaded areas represent the corresponding 95% confidence intervals.P values for the overall and nonlinear associations are displayed in each panel.

Abbreviations: SBP, systolic blood pressure; LDL-C, low-density lipoprotein cholesterol; HbA1c, glycated hemoglobin; OR, odds ratio; CI, confidence interval.

2

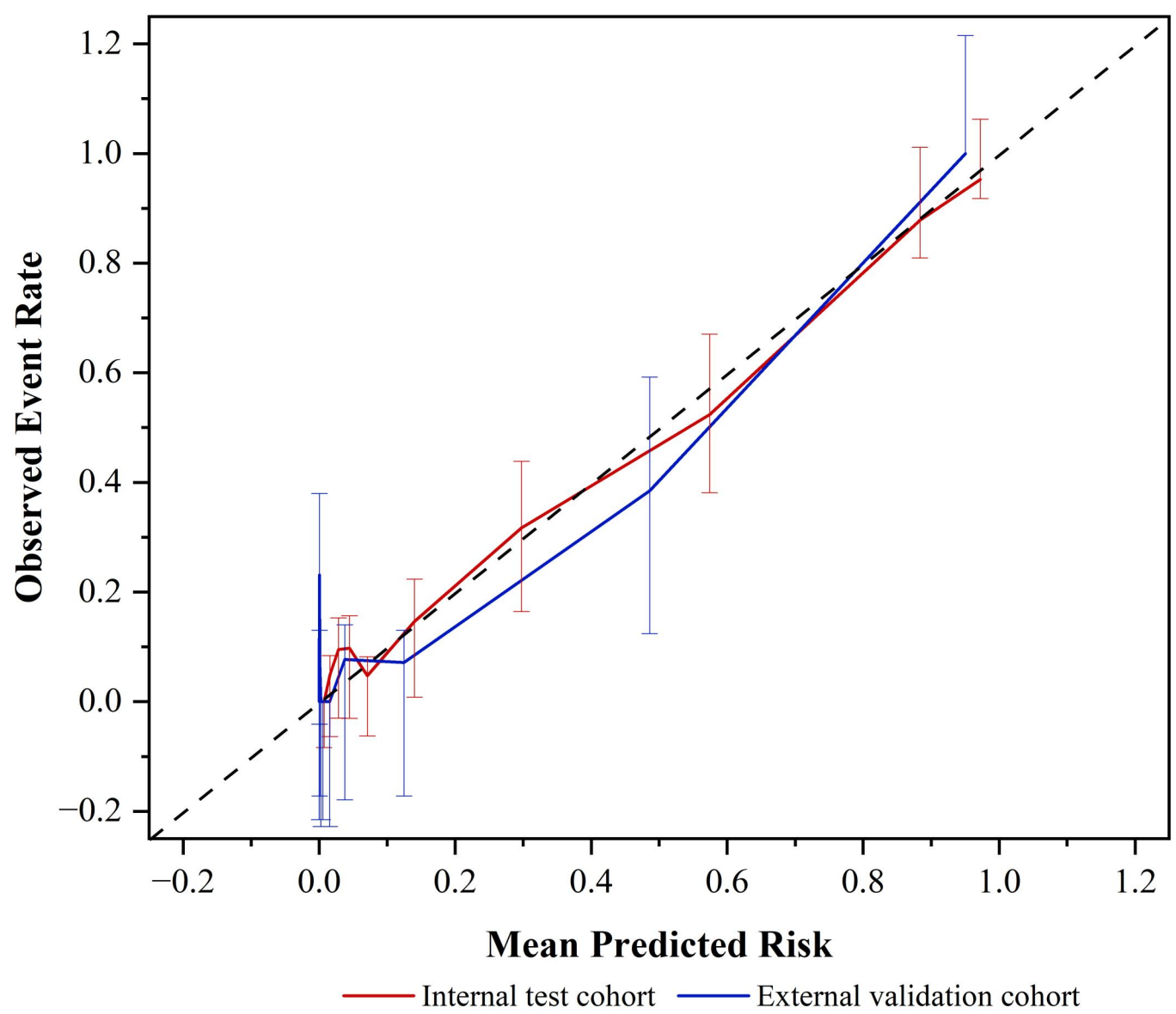

Supplementary Figure 2. Calibration plots of the multimodal model in the internal test and external validation sets.

Notes: Calibration plots of the multimodal model in the internal test set and the external validation set. The diagonal reference line represents perfect calibration, whereas the model calibration curve represents the agreement between the predicted probabilities and observed outcomes. Greater proximity to the diagonal reference line indicates better calibration.
